# Provincial Medical and Surgical Association

**Published:** 1839-07

**Authors:** 


### PROVINCIAL MEDICAL AND SURGICAL ASSOCIATION.

It is gratifying to observe the progressive growth, in extent and usefulness, of this excellent society. The members now amount to 1,200. It comprehends six district branches, all of which are in a flourishing condition. These district branches are local *reunions*, established for the purpose of promoting the general objects of the Association, and furnishing some substitute for the general meetings to those members who cannot follow the latter in their annual migrations. The *Bath District Branch* held its third annual meeting at Bath, on the 6th of June, and was well attended. Mr. Soden was president. The members met in the forenoon, at the Literary and Scientific Institution, and afterwards dined together. Dr. Barlow, the secretary, read a very satisfactory report, from which it appeared that the number of members amounts to 84. The *Southern District Branch* held its annual meeting at Portsmouth, on the 13th of June, and was attended by members from Salisbury, Winchester, Southampton, Chichester, and all the other towns within the district; the number present being upwards of 80, including several medical gentlemen, not members. Dr. Quarrier was president. The meeting was held in the Guildhall, and the members afterwards dined together. Several interesting communications were made to the society, and a most admirable report on the state of surgery, during the past year, was read by Mr. Newnham, of Farnham, and which he was unanimously requested to publish, for the benefit of the profession generally. This request, we are happy to say, Mr. Newnham and his coadjutors, Mr. Wickham, of Winchester, and Mr. Salter, of Poole, complied with; and the publication of the report has accordingly taken place: the profits to be given to *The Benevolent Fund* of the Association. The next meeting of the branch was fixed for Chichester, and Dr. Forbes was appointed president.

The seventh anniversary meeting of the Association will be held at Liverpool, on the 24th and 25th of the present month (July), under the presidency of Dr. Jeffreys. It is expected to be the fullest that has yet taken place, not merely from the increased number of the members of the Association, and populousness of the place of meeting, but from the great importance of some of the subjects to be discussed; more particularly those of *vaccination* and *medical reform*. This meeting will also possess an attraction of a peculiar kind, which, although having more of a personal than a professional character, cannot fail to interest every one belonging to the association; we may indeed say, every lover of his profession, and every admirer of what is excellent and honorable in its members. We refer to the presentation to his family of a PORTRAIT OF DR. HASTINGS, by the members of the association. We cannot better advert to this subject than by using the words of Dr. Barlow, at the late meeting at Bath. "The District Council (he said) wish to advert to the slight tribute of gratitude offered to the respected and estimable founder of the association, Dr. Hastings. The portrait of him designed as a present to his family, is completed; and the engravings are expected to be ready for delivery to the subscribers at the ensuing general meeting. If the claims of Dr. Hastings on the gratitude of the association were to be estimated at their real amount, no recompence could adequately remunerate the services which he has rendered, and continues to render. For these, his best reward must be derived from the heart-cheering consciousness of talents and energies ably and zealously devoted to a most praiseworthy and beneficial end. To those for whom he has laboured, it cannot but be gratifying to possess a memorial of one to whom they are so deeply indebted; and no memento could be more appropriate or interesting than one which will keep continually in view the intelligent and expressive countenance of their benefactor and friend."

